# Needle Guide Friction Reduction Utilizing Contact Surface Structure

**Published:** 2024-06-25

**Authors:** Isaac Elijah, Sang-Eun Song

**Affiliations:** 1 Charles E. Schmidt College of Medicine, Florida Atlantic University, Boca Raton, FL, USA; 2 Department of Mechanical Engineering, University of Akron, Akron, OH, USA

**Keywords:** Prostate biopsy, Core biopsy, Needle guide, Insertion friction

## Abstract

Prostate cancer ranks as the second most common cause of cancer-related mortality among males. A template guided core biopsy procedure is an essential component of prostate cancer diagnosis. Various factors influence the quality of the histological sample that is produced from the biopsy, including tissue fragmentation, needle insertion speed, needle deflection, and sheath cutting speed. In instances where a biopsy core fails to meet established clinical standards, an additional core extraction is necessary, resulting in heightened patient discomfort and an increased risk of infection. The objective of this study is to examine alternative configurations for the needle guide in order to optimize for the reduction of needle guide frictional forces while providing adequate guidance towards a target. A simulation of this interaction using different configurations of the needle guide was designed using SIMULA Abaqus, and a Finite Element Analysis was performed to observe the friction force between the needle and guide. The results showed that configurations with contact points oriented parallel to the direction of insertion exhibited the least recorded frictional forces. The study demonstrated that configurations which provided decreased contact surface compared to the control needle guide resulted in lower friction force. These findings advocate for the optimization of the conventional biopsy needle guide to reduce the contact area between the needle and guide inner surface, potentially reducing the number of passes required to obtain a histologically viable core specimen and mitigating the risk of patient infection.

## INTRODUCTION

### Prostate Cancer and Core Biopsy

Prostate cancer stands as the most common cancerous malignancy among males, projected to represent an estimated 29% of cancer diagnoses in 2024. In the United States, an estimated 1 in 8 males were anticipated to receive a diagnosis of prostate cancer, with an associated mortality rate of 11%, ranking it as the second-leading cause of cancer-related death among males [1]. The average age of death due to prostate cancer is 80 years old, highlighting the importance of periodic screening after age 55 [2].

Following an abnormal digital rectal examination (DRE) or prostate-specific antigen (PSA) level, a physician may recommend performing a biopsy. The biopsy procedure typically involves a specialized tissue sampling mechanism comprising an inner needle equipped with a recess for isolating a specimen and a deployment mechanism (such as spring-loaded or manual) that encases the needle with an outer cutting sheath. This tissue cutting apparatus operates in two distinct phases: an initial phase characterized by the accumulation of needle force until it reaches the threshold required to separate the tissue, followed by a secondary phase where needle force stabilizes after tissue separation has occurred [3]. The diverse array of forces exerted on the needle during each stage can induce deflection and other forms of deviation, ultimately influencing the accuracy and quality of the biopsy sample.

To ensure accurate evaluation, the biopsy specimen must possess specific histological characteristics that render it suitable for analysis. The specimens are typically produced as cylindrical cores of the target tissue, shaped by the cutting sheath. The specificity of the sample and the false-negative rate of cancer diagnosis it yields are largely contingent upon the volume of the sampled tissue [4]. Given the correlation of volume with length, a biopsy core of greater length and diameter is preferable. Fragmentation of the biopsy sample poses a concern, as it impedes the ability to draw conclusive data regarding the region’s physiology. When an optimal sample is obtained, it undergoes analysis using a standardized scale known as the Gleason Score. This scoring system, ranging from 1 to 5, is utilized to categorize the progression of cancer within the tissue [5].

### Needle Guide and Insertion Speed

The accuracy of core biopsy needle insertion location is influenced by the interaction between the needle guide and the needle insertion mechanism. In core prostate biopsies, adhering to a template may facilitate more uniform and evenly distributed sampling of the prostate compared to the traditional freehand biopsy approach, potentially reducing the risk of false-negative biopsies [6]. However, the use of a grid or template has been associated with certain drawbacks. For instance, a tighter guide hole leading to increased friction can impede the speed of the needle insertion and sheath cutting, potentially resulting in poor sampling. Nonetheless, despite variations in insertion speed, an underlying enhancement in the precision and accuracy of needle insertion is generally observed [7]. The tightness of the stabilizing agent to the needle can impact the correlation between grid coordinates and degrees of rotation during needle insertion, introducing the possibility of technical and equipment-related errors. Consequently, meticulous technique remains crucial even when employing a template for insertion, as it does not guarantee reproducible needle placement [8].

The speed at which the needle is inserted into the target tissue significantly impacts the histological quality of the final sample, primarily through its influence on dynamic soft tissue deformation. Experimental analysis has demonstrated that maximum tissue deformation occurs during the initial insertion of the needle, resulting in the obscuring of the target lesion at the point of contact between the tissue and needle. However, beyond this point, an increase in insertion speed leads to a reduction in deformation owing to the generation of kinetic and viscous energies. This decrease in targeting error results in a biopsy sample of greater histological value [9].

During needle insertion, the contact between the needle surface and tissue generates friction stresses. The average friction stress along the needle-tissue interface decreases with increasing insertion speed [10]. Enhanced needle insertion speed not only reduces the force required during the initial phase of the tissue-cutting procedure but also minimizes tissue displacement during the subsequent phase. Moreover, high-speed needle insertion contributes to improved histological sample quality by producing biopsy cores with a higher mean molecular weight [11].

When a biopsy needle is inserted at a desired location, the inner needle is further inserted by triggering the first step of a spring loaded deployment mechanism or manually. Once the target tissue is within the sampling space, the second trigger is activated, causing the outer sheath to rapidly advance and cut the tissue with its sharp edge [12]. If the speed is not sufficient, the sheath may push the tissue instead of cutting resulting in poor or no sample. Since the inserted sheath comes into direct contact with both the tissue and the needle guide, the resulting friction can slow down the deployment speed. Assuming that the tissue friction has already been accounted for by the device manufacturer, the additional friction caused by the needle guide can further reduce speed. Therefore, minimizing this friction is essential to ensure the biopsy device performs as designed.

### Proposed Approach

In order to enhance the efficiency of prostate core biopsy procedures and consistently produce high-quality samples, it is imperative to minimize factors that obstruct needle insertion and sheath deployment. Given the significant role of friction in affecting insertion speed, tissue displacement, cutting speed, and the likelihood of core fragmentation, this study aims to mitigate friction force along the interface between the template guide and the outer surface of the sheath. While previous studies have explored methods to reduce friction within the biopsy system - such as sharpening the beveled needle tip and polishing the inner surface of the needle to minimize roughness - there appears to be little data investigating the interaction between the needle template and the needle itself. Despite the widespread adoption of template-guided biopsies over decades, the needle guide has remained relatively unchanged and largely overlooked as a potential source of friction in the system. This study seeks to address this disparity by simulating the interaction between these two components and evaluating the extent to which altering the configuration of the internal surface of the template can reduce friction force.

## MATERIALS AND METHODS

### Guide Design and Material

The varied patterns of the internal surface of the biopsy template were devised to assess the impact that the number and orientation of contact points relative to the direction of needle insertion would have on friction force. In contrast to a smooth inner surface, the guides were structured with semicircular extrusions aligned in the direction of the specified condition. To explore a broad spectrum of friction reduction scenarios, models were tested with three and five of these internal patterns, oriented either parallel or perpendicular to the direction of needle insertion. Additionally, a control condition was incorporated, featuring no internal surface pattern to represent the current industry template guide design. Considering the simulation software’s limitations, it was anticipated that simulating the needle with the exact diameter as the guide it entered would not generate sufficient contact for measurable friction force values. Therefore, a null control condition was initially conducted with equal outer and internal diameters of the needle and guide. Should this condition yield undetectable results, the needle diameter would be increased in subsequent conditions to generate a minimum detectable overlap between the two simulation components.

Maintaining MRI compatibility, as per applicable medical protocol for prostate biopsies [13], was paramount in this simulated study. Consequently, the biopsy needle was modeled from titanium, while nylon was chosen for the template guide due to its low friction, nontoxic properties, and suitability for injection molded plastics - an ideal method for large-scale production [14]. The nylon template guide design allows for the exchange of the internal guide component. Over time, plastic deformation may occur, potentially enabling nonaxial needle movement during insertion. Hence, this mechanism facilitates the straightforward replacement of worn guides with new ones, ensuring that the original efficacy is maintained.

Finally, adjustments were made to the needle component of the simulation model to align with the study’s focus on friction between the needle and the template guide. The needle tip was configured as a short cone rather than a typical beveled tip as the friction study focuses on needle body contact not the tip, and the needle was depicted as a solid cylinder instead of a hollow tube, given that the primary interaction would result from nylon cylinder deformation. Considering that titanium metal is over fifty times stronger than nylon [15,16], any surface deformation of the titanium needle would be negligible and thus simulated as solid.

### Simulation Parameters

The SIMULA Abaqus software was used to perform a finite element analysis on a simulation of a biopsy needle passing through a guide. Several constraints were placed on the study. Firstly, the simulation will take place under dry friction conditions, meaning the application of lubricants or similar friction-reducing substances will not be employed. The dimensions of the biopsy needle will follow the specifications of an 18-guage biopsy needle. Needles of varying dimensions will produce different results.

Because Abaqus inputs do not specify units, a consistent set of units is required to be utilized across the entire simulation setup i.e., International System of Units (SI). In this simulation, the following units were used: length was designated in mm, force was designated in Newtons (N), time was designated in seconds (s), stress in MPa (N/mm2), and energy in milli Joules (mJ).

The simulation consisted of two parts: first, a short, narrow, solid cylinder with a conical tip representing the outer sheath of the biopsy needle. The template guides were represented by hollow cylinders with varying internal surface patterns according to the condition. In order to determine the diameter of the needle, a null control condition was performed in which the radius of the needle was set equal to the radius of a guide with no internal surface pattern. If the interaction between the guide and needle is approximately zero, this will indicate a need to increase the diameter of the needle for the purpose of simulation in order to produce quantifiable results. The outer diameter of the needle and the inner diameter of the guide were both dimensioned to 1.27mm (18 gauge needle) for the null control condition, and the radius of the needle will be increased to 0.6395mm if the null condition produces a positive result. For conditions employing an internal surface pattern, the pattern was modeled as semicircular ridges with a radius of 0.11mm and running direction specified by the condition. All needle guides were 1mm in length.

In all conditions, the material of the needle was titanium and the material of the guide was nylon. The parts that both of these materials were applied to were solid and homogeneous. The titanium had a Young’s Modulus of 113 GPa and a Poisson’s ratio of 0.3. The nylon had only elastic material behavior, with a Young’s Modulus of 2700 MPa and a Poisson’s ratio of 0.39. The Nylon material also had a Yield Stress of 45 MPa.

### Study Conditions

The following five conditions were tested, reflecting the range of internal surface patterns of the needle guide for this study: Null Control, which had no internal surface pattern; Control, which had no internal surface pattern; Perpendicular 5-point contact, which had five ridges running perpendicular to the direction of insertion; Perpendicular 3-point contact, which had three ridges running perpendicular to the direction of insertion; and Parallel 3-point contact, with three ridges running parallel with the direction of insertion. Each of these conditions were placed in a separate model in Abaqus along with the needle, a total of two parts per model.

All conditions were assembled with the direction of insertion going towards the positive y-axis direction, and those that contained internal ridges had them uniformly distributed along the inner surface of the guide. All guides had a height of 1mm, and the distance between the innermost points of contact and the center of the cylinder were constantly dimensioned at 0.635mm to reflect the 1.27mm outer diameter of an 18-guage needle.

For the two needle guide conditions which had the internal pattern running perpendicular to the direction of needle insertion, the 360° revolution around the y-axis was used to create the cylinder. These conditions were initially sketched as an outline of the perimeter of the guide in one quadrant, and the revolution around the y-axis developed it into a three-dimensional part. For the two needle guide conditions in which the internal ridges were running parallel with the direction of needle insertion, an extrusion was used. These parts were initially sketched as a smaller circle inscribed in a larger circle with the ridges part of the internal circle, and an extrusion of a certain width (i.e., the height of the guide, 1mm) developed the condition into a three-dimensional part. Several constraints were used in the sketching of the guides. The internal ridges were constrained to be equal in radius and equally spaced apart from one another across the 1mm length of the guide. The outer and inner surfaces were parallel with one another, and the lower and upper surfaces of the guide were parallel with one another.

### Material Properties

The needle guide was assigned the plastic material Nylon. This material has isotropic elastic properties without compression or tension capabilities. The Young’s Modulus was designated to be 2700 MPa (or 2.7GPa) and the Poisson’s ratio 0.39, and the material was solid and homogeneous. The needle was assigned to be titanium with only isotropic elastic properties as well. The Young’s Modulus was designated to be 113,000 MPa (equivalent to 113 GPa), nearly fifty times that of nylon, and a Poisson’s ratio of 0.3.

The following tab to be completed was the Assembly tab. In this tab, the parts of a model were placed in a three- dimensional space in the desired orientation. For all of the conditions, the direction of insertion was oriented as the positive y-axis direction, meaning the needle would move upward through the needle guide. The entry point of the guide was placed at 0.25mm above the origin, and the tip of the needle was placed at the origin facing upward to move through the guide. A reference point was also added at 0.25mm above the origin along the y-axis for later use.

The next tab to be completed was the Step tab. This tab contains options for different analysis types and output controls. In this tab, a new set and history output were created. The set is used to associate the dynamic part of the model with the refence point, and a history output of certain data is generated according to what forces that reference point experienced during the simulation. Under Tools, the reference point at 0.25mm above origin was created as a new set. Then the History Output Manager was used to create a new History Output that is correlated not with the entire model, but with the new reference point set that was just created. The variables that were selected to be outputted were the reactive forces in all three dimensions (RF1, RF2, and RF3), though the major force of interest is RF2 because this represents the friction force that the needle experiences as a result of entering through the guide.

The guide will slightly deform upon entry of the needle, and as a result produce a small output of force in the x- and z-dimensions, but these values were expected to be approximately zero. Within the Step tab, the two interfaces that would be actualized during the simulation were specified. An initial step was created in which no propagation of the needle takes place, but the correct orientation which was designed in the assembly was saved. A propagation step was also specified (called Step-1), where incrementation was set; the initial increment was 0.05, the minimum was 1E- 008, and the maximum was 1. The maximum number of increments was set to be 10,000, and the Nlgeom was turned on to account for nonlinear stress and strain in the simulation.

### Surface Interaction

For this study, two interactions were designated: an initial and a Step-1 propagation interaction. The initial interaction type was a standard surface-to-surface contact in which the master surface was the entire outer surface of the needle and the slave surface was the inner surface of the guide, including the ridges and the non-contact areas. Three-dimensional surfaces were not smoothed. Also, the reference point was coupled to the entire outer surface of the needle. This was done so that the reference point can be made to move under certain boundary conditions and allow the entire needle to move accordingly.

Boundary conditions are designated to fix certain aspects of the model and apply an initial force in the desired direction. Three boundary conditions were used for this simulation. Firstly, a single point of the guide was constrained to be fixed in place; the boundary condition type was Symmetry/Antisymmetry/Encastre. This was done so that the needle guide would not move when contact with the needle was established. The second boundary condition type was also Symmetry/Antisymmetry/Encastre. Here, the bottom surface of the needle guide (the face pointing in the direction of the needle) was restricted to allow no movement in the y-direction. By doing this, the cylinder will be allowed to expand and contract when in contact with the needle, but the combination of the fixed point and the lack of vertical movement of the bottom surface will fix the entire cylinder in place. The third boundary condition type was Displacement/Rotation and was placed on Step-1. This third boundary condition moved the reference point, and therefore the entire needle, 2.0mm in the U2 direction (upward, positive y-axis).

Finally, regarding mesh, the needle guide was designated to have a seed size of 0.01, and each condition had a different number of total elements due to the difference in shape. The needle was given a seed size of 0.02 equaling approximately 1300 total elements.

**Figure 1:**
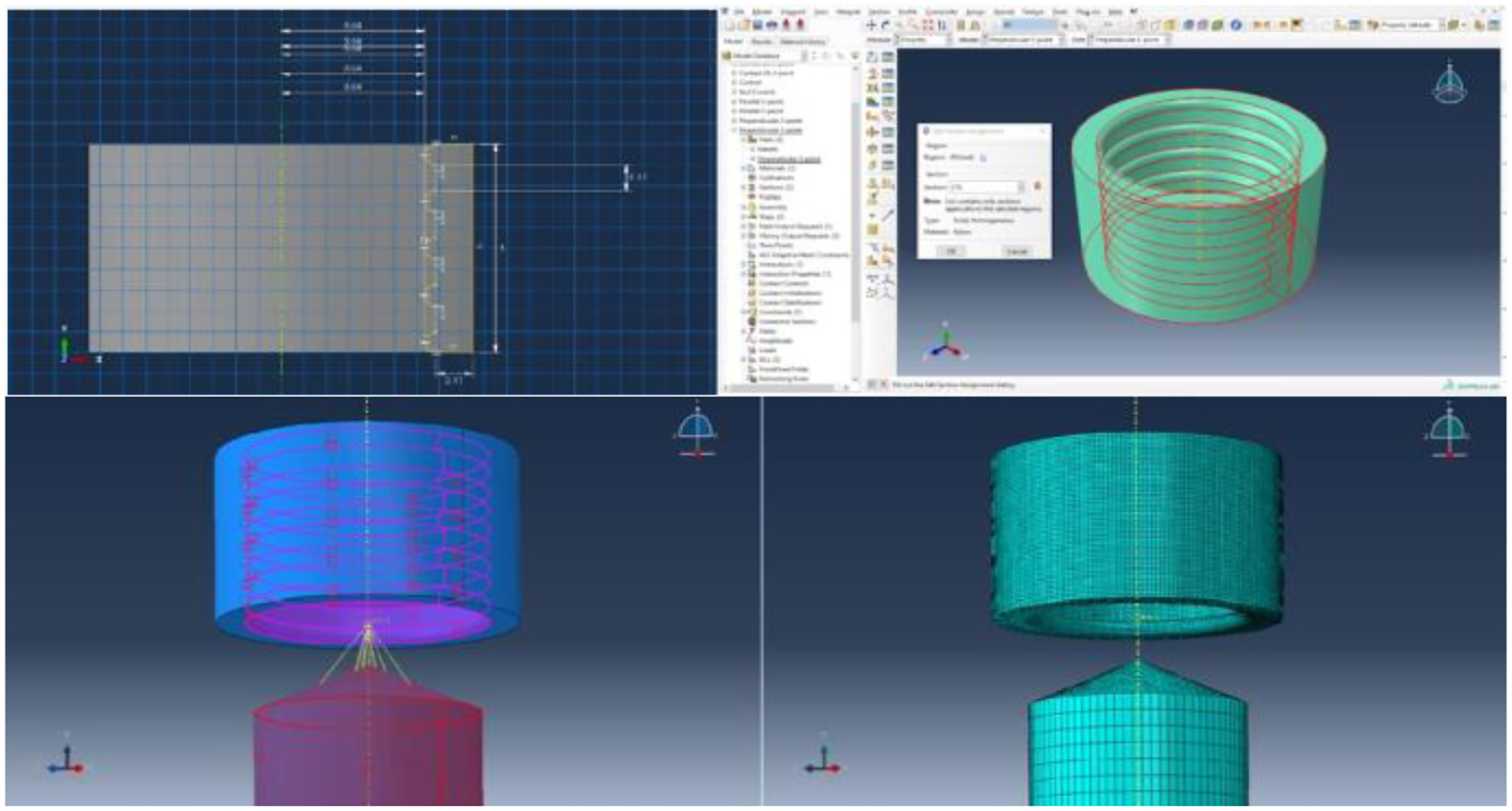
Development of the Perpendicular 5-point condition. First, the dimensions of the two components of the simulation were outlined, including the template (upper left) and the needle. These components were converted to a three-dimensional part by applying a 360° rotation around the y-axis. The Nylon material properties were then applied to the template (upper right), while the titanium material properties were applied to the needle using the Properties tab. The two parts were oriented along the y-axis using the Assembly tab, and the Load tab was used to fix the template in its position and set a displacement path for the needle of 2.0mm in the positive y-axis direction (lower left). Finally, the Mesh tab was used to apply a grid to each component of the simulation to distinguish data points at which the deformation and friction force can be measured (lower right). The desired output variables (i.e., the reaction forces in three dimensions) were specified in the Step tab, and the simulation was run in the Job tab

Figure 1 illustrates an overview of this process for the Perpendicular 5-point condition, with the complete methodology for each condition. After the simulation was completed, the Visualization tab showed how different elements of the mesh experienced different deformation, indicated by a color gradient. A plot of the friction force versus time in the U1, U2, and U3 directions (i.e., RF1, RF2, and RF3, respectively) were generated for each model for comparison.

## RESULTS

### Mathematical Correlation

Before carrying out a simulation, the physical scenario which the simulation claims to represent needs to be formulated into an equation that can be used to produce expected values. By doing this, the accuracy of the simulation to a physical circumstance can be assessed according to the degree of similarity between the expected and actual results.

The system that is represented by the simulation is one in which a cylindrical object is experiencing only internal pressure in the outward direction, reflecting the needle moving through the guide and causing deformation. By relating and simplifying the fundamental equations in Table 1, we can derive Equation 1 to mathematically represent the simulation. The final derived equation is:

(1) $$F=\frac{4\mu \cdot \Delta \mu \cdot t\cdot E\cdot A}{d_{i}(d_{i}+t)}(1+f_{A})$$

demonstrating the friction force along the internal surface of a cylindrical structure as a secondary component passes through. The input and output parameters of the equation were incorporated into a function within an Excel spreadsheet, with values specified for each input variable for both the control condition and experimental conditions. For the control condition, an inserted contact distance of 1mm was designated because the needle contacts the entire inner surface of the guide for the duration of the simulated insertion. A lower value was estimated for the experimental conditions since the needle will not be contacting the entire inner surface of the guide, but only a fraction of it. Additionally, the area correction factor was adjusted to be smaller for the experimental conditions compared to the control condition, considering the reduced number of contact points and total contact area in the experimental conditions.

The guide diameter and thickness, and the overlap between the two parts were constant across all conditions. As per the derived equation, the control condition was anticipated to yield a relative friction force of approximately 8.43N, while the experimental conditions were estimated to produce a friction force of around 3.09N. These values serve as rough estimates of the friction force and are expected to vary in magnitude during simulation, particularly for the experimental conditions. This variability arises from the utilization of a single equation to correlate all the different experimental conditions and their associated variations in contact surfaces (i.e., number and orientation of contact points). To assess the validity of the simulation, a margin of 50% for the average of the friction forces of the experimental conditions (i.e., 3.09 ± 1.55) and a margin of 30% for the control condition (i.e., 8.43 ± 2.53) are employed. These margins provide a range within which the actual friction forces are expected to fall, thereby enabling evaluation of the simulation’s accuracy.

The results derived from the equation indicate that a decrease in contact area corresponds to a reduction in friction force, thus offering theoretical and mathematical support for the simulation. Consequently, the simulation’s validation will be contingent upon its ability to replicate this trend. All dimensions, values, and constants in the derived equation precisely reflect the parameters within the simulation itself to ensure consistency between the theoretical framework and the practical implementation.

### Null Control Results

**Figure 2:**
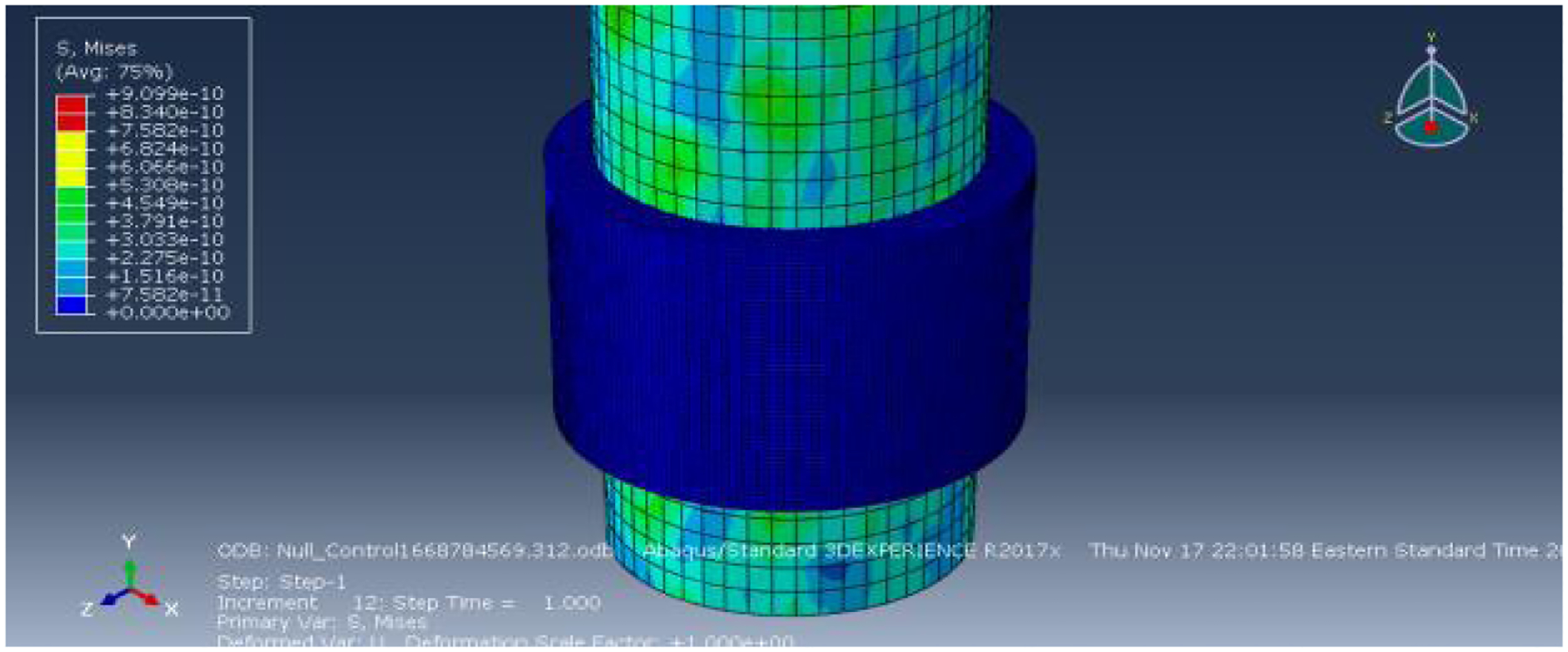
Deformation map of the Null Control condition.

The first simulated condition was the Null Control condition, where both the outer diameter of the needle and the inner diameter of the guide were set to 1.27mm to assess the necessity of simulating manual overlap. The color deformation map and plot of the resulting friction force (RF2) are presented below. As depicted in the deformation map (Figure 2), there is no observable deformation of the needle guide, with all the seeds remaining at their initial baseline values and associated representative color. The needle experienced some reactive force, not attributed to interaction with another surface, but rather due to its movement upwards through space. The plot of the friction force over the simulation duration further confirms the apparent lack of interaction between the two surfaces.

**Figure 3:**
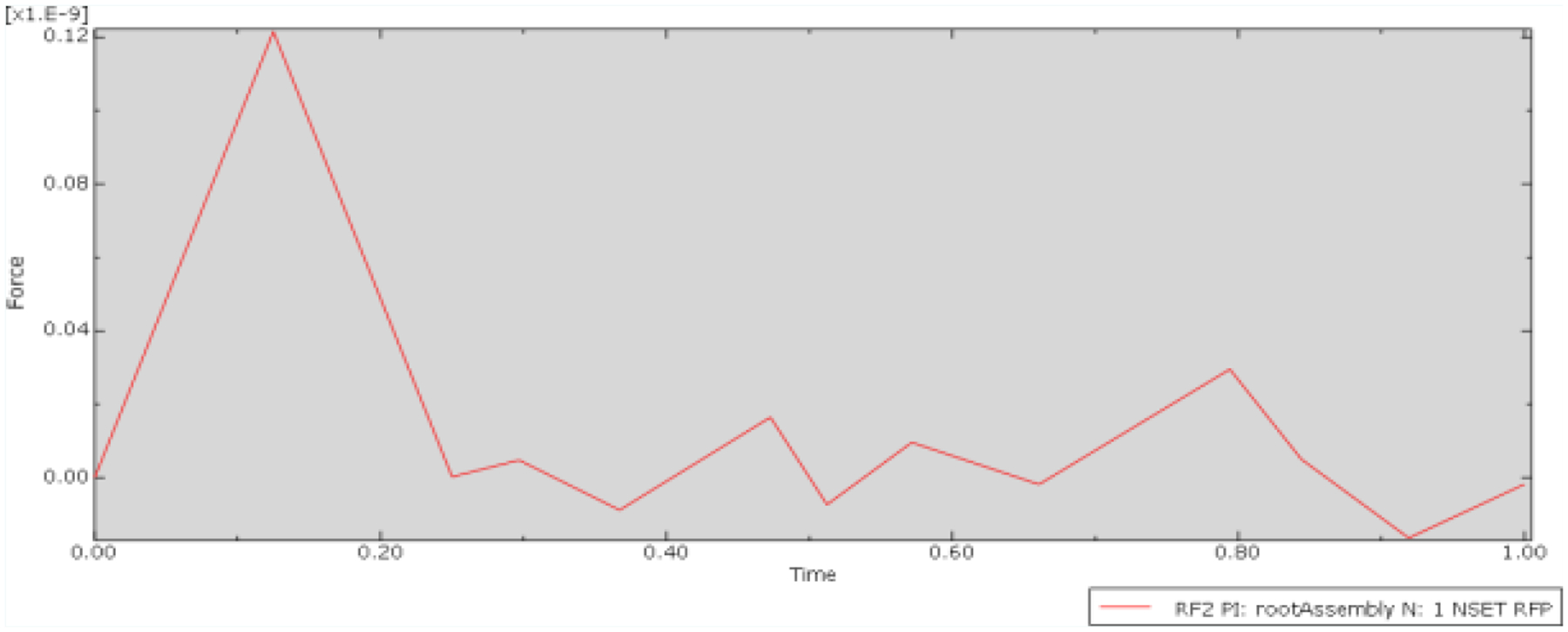
Plot of friction force versus time for the Null Control condition

As indicated in the upper left corner of the friction force plot (Figure 3), the scale for the reactive forces was at an order of magnitude of 10^−9N. This magnitude signifies an exceedingly small friction force, which falls well below the predicted values outlined in the derived equation. Consequently, it effectively signifies negligible interaction between the needle and the guide. Furthermore, the oscillating trend observed in the plot suggests that the force experienced was predominantly a result of motion rather than contact with the other surface. This contrasts with the anticipated trend, where one would expect to observe a gradual increase in friction force before reaching a maximum value and leveling off. In light of these findings, the subsequent experimental conditions were conducted with a slightly larger diameter needle to induce an overlap between the two parts. This adjustment was necessary to ensure appropriate comparison between the conditions and to elicit the expected and realistic interaction between the needle and the guide.

**Figure 4:**
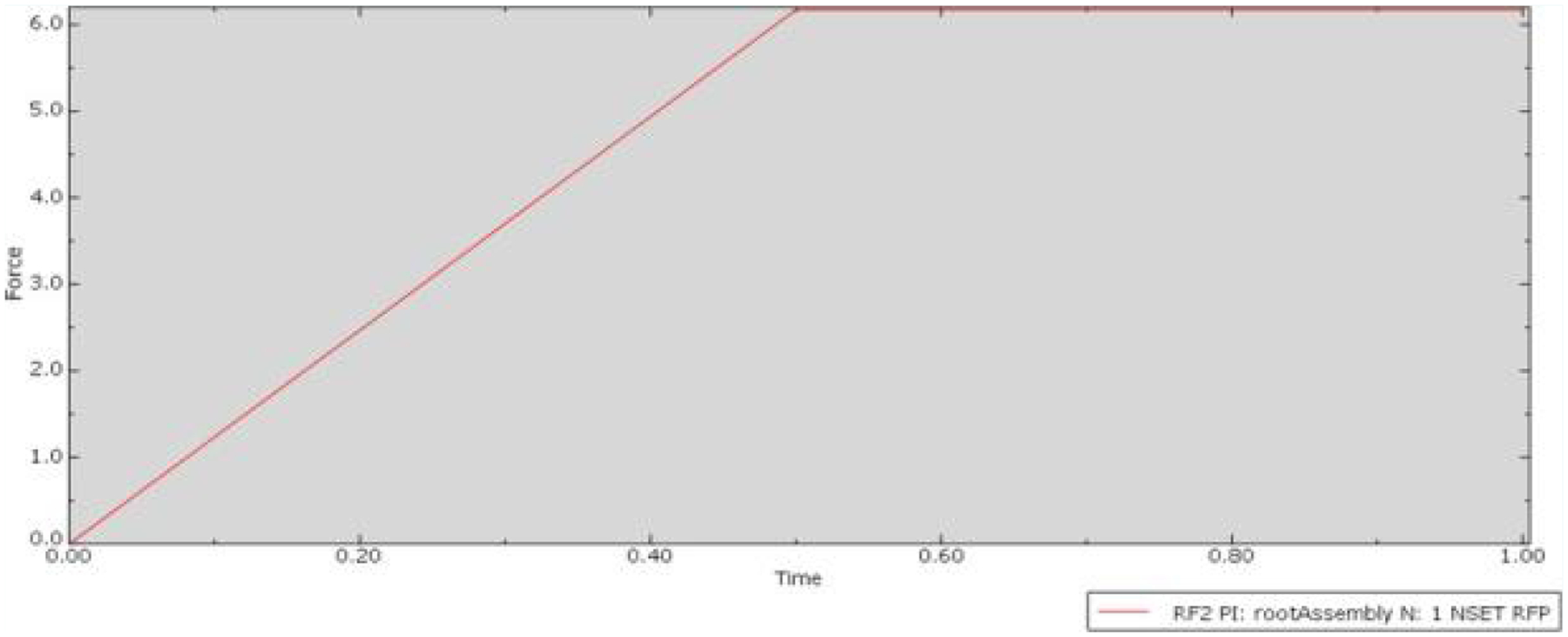
Plot of friction force versus time for the Control condition.

In the Control condition and the experimental conditions, an induced overlap of 0.0045mm was included. The deformation map (Figure 4), showed the anticipated outcomes, with deformation predominantly occurring in the nylon needle guide rather than the significantly harder titanium needle. The distribution of deformation indicates that the highest force was experienced at the interface between the guide and the needle, which decreased gradually with distance from the contact points.

### Perpendicular Ridge Surface

**Figure 5:**
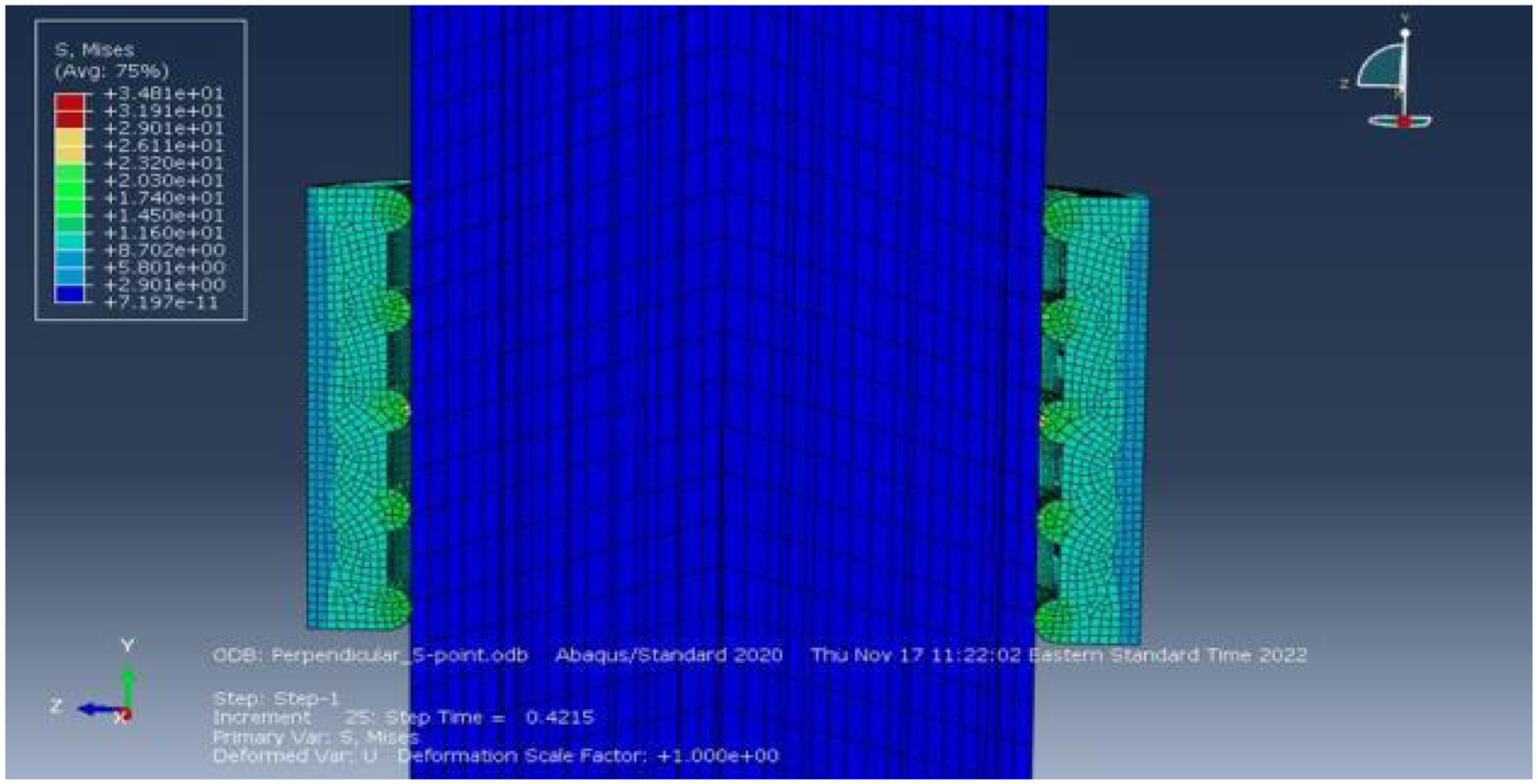
Deformation map of the Perpendicular 5-point condition

**Figure 6:**
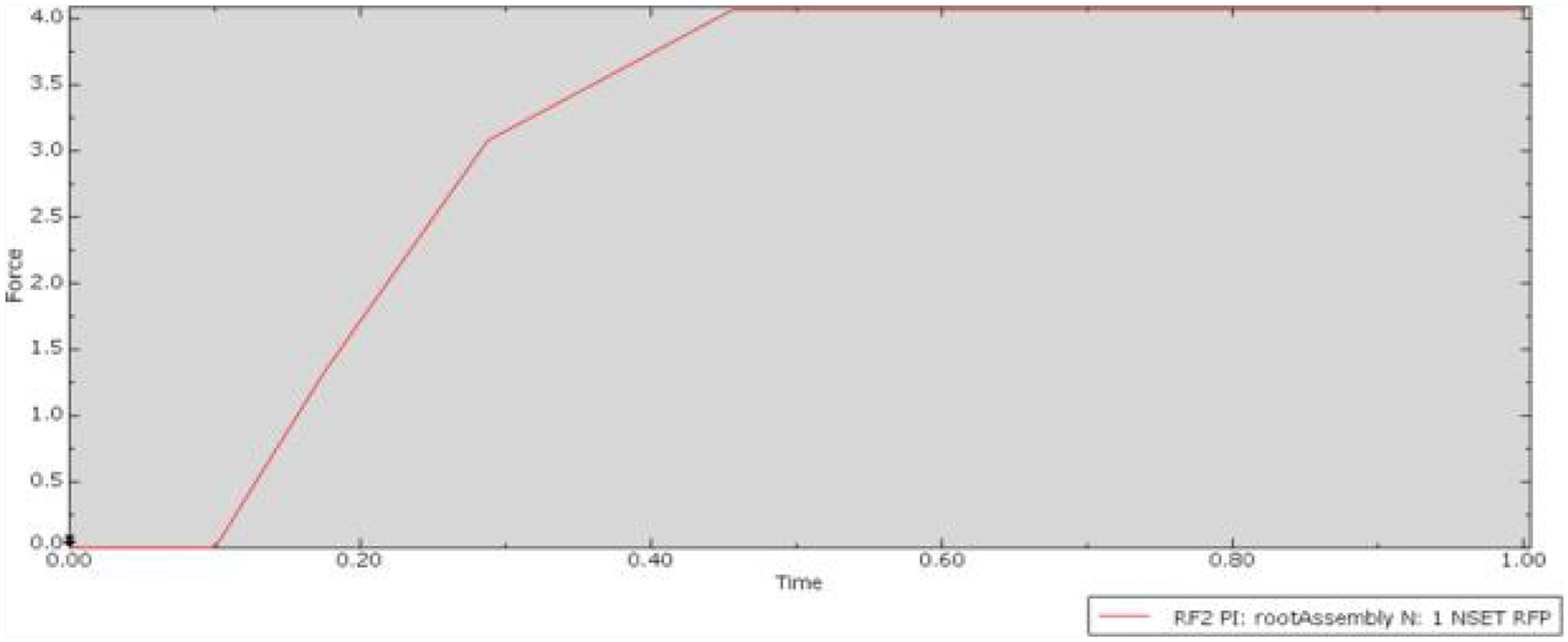
Plot of friction force versus time for the Perpendicular 5-point condition

The conditions featuring ridges oriented perpendicularly to the direction of insertion also reflected to the theoretical framework outlined by the derived equation. The deformation map of the Perpendicular 5-point condition (Figure 5), exhibited an anticipated distribution of friction force, with the contact points experiencing the largest deformation and the reactive force dispersing outward from these points. The corresponding plot for this condition (Figure 6), displayed a peak friction force of approximately 4.0N. This indicates a reduction of 33% in friction force between the two surfaces compared to the Control condition.

**Figure 7:**
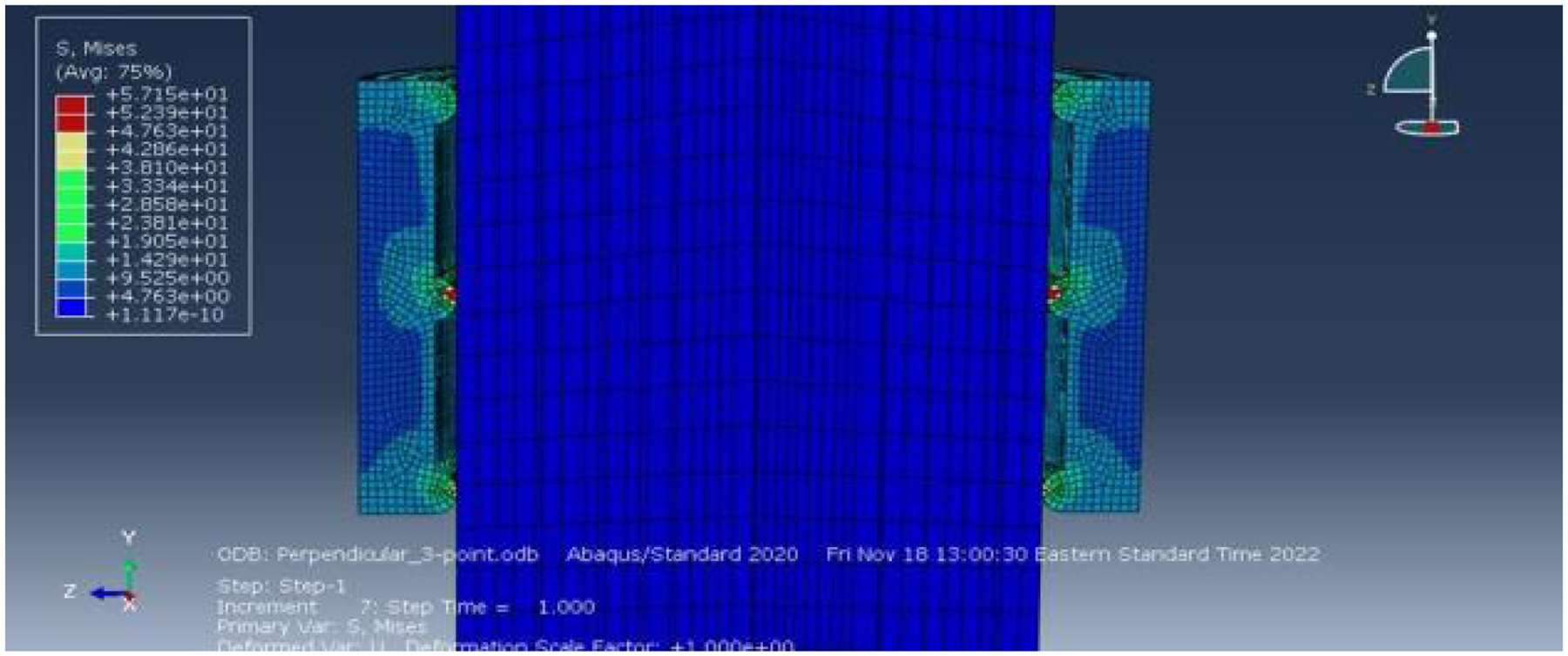
Deformation map of the Perpendicular 3-point condition.

**Figure 8:**
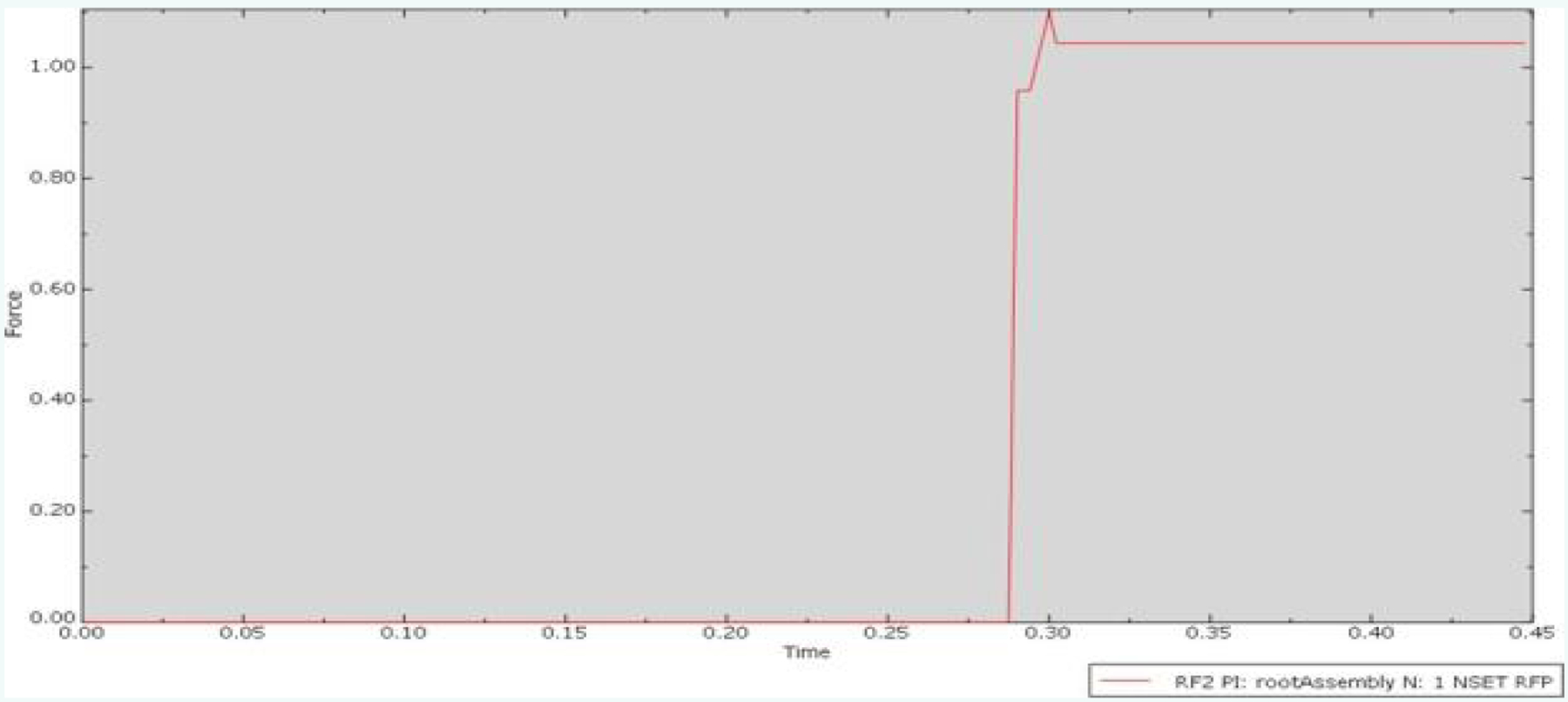
Plot of friction force versus time for the Perpendicular 3-point condition.

The Perpendicular 3-point condition exhibited a lower peak friction force compared to the Perpendicular 5-point condition. The deformation map (Figure 7), demonstrated a reasonable distribution of stress on the needle guide, with minimal deformation observed in the needle itself. However, the plot for the Perpendicular 3-point condition (Figure 8), indicated a peak friction force of approximately 1.2N, representing a substantial reduction of nearly 80% compared to the Control condition. This observation aligns with the predictions of the derived equation, which suggests that a decrease in contact area leads to a lower associated friction force in the interaction.

### Parallel Ridge Surface

Given that the Perpendicular conditions exhibited the anticipated trend outlined by the derived equation, which suggests that a lower contact area and fewer contact points result in reduced friction force, the subsequent investigation focused on evaluating the impact of ridge orientation on friction force. Consequently, a 3-point condition was specifically conducted with the Parallel orientation, as implementing a Parallel 5-point condition would be redundant.

**Figure 9:**
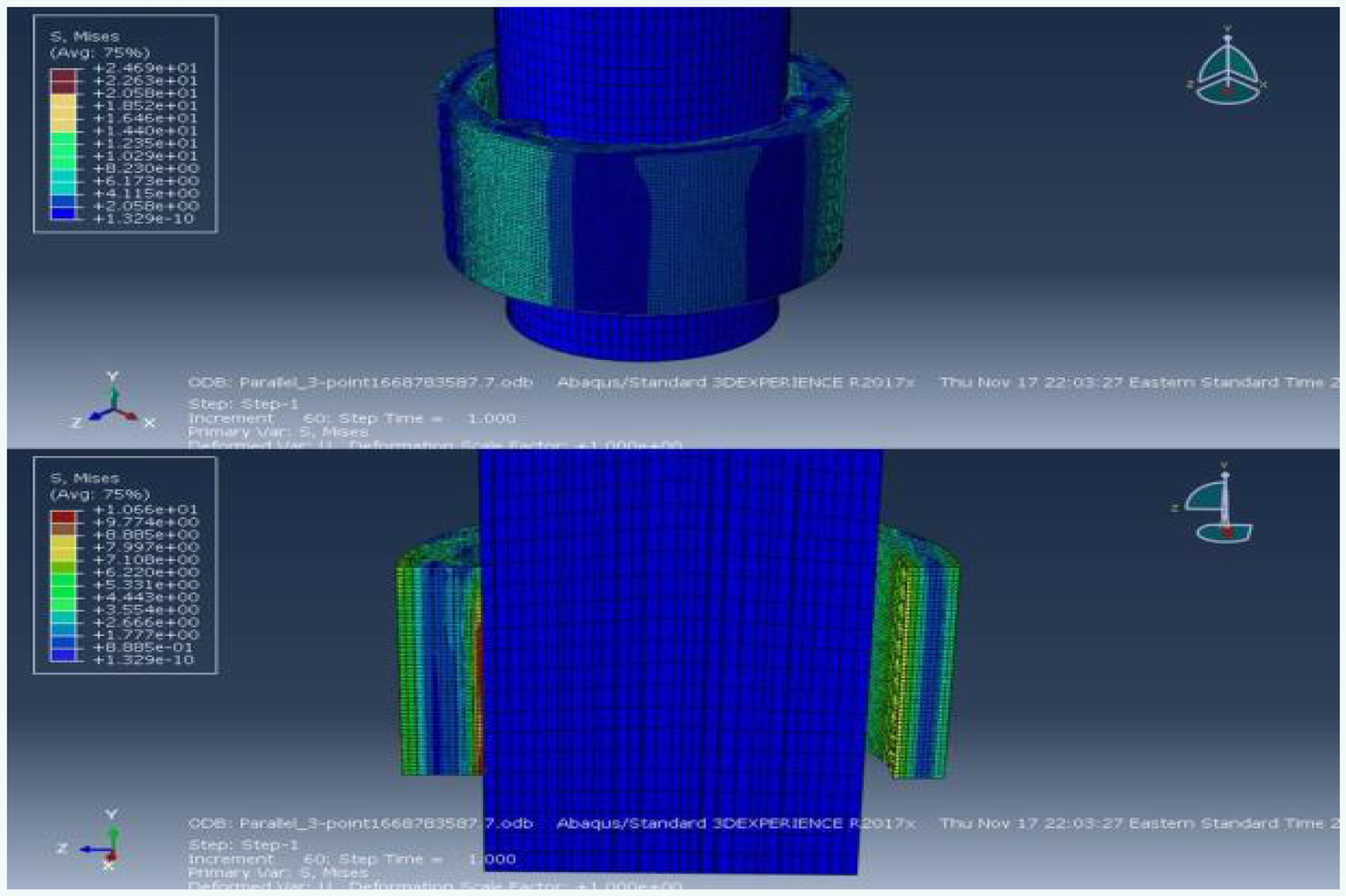
Deformation map of the Parallel 3-point condition.

**Figure 10:**
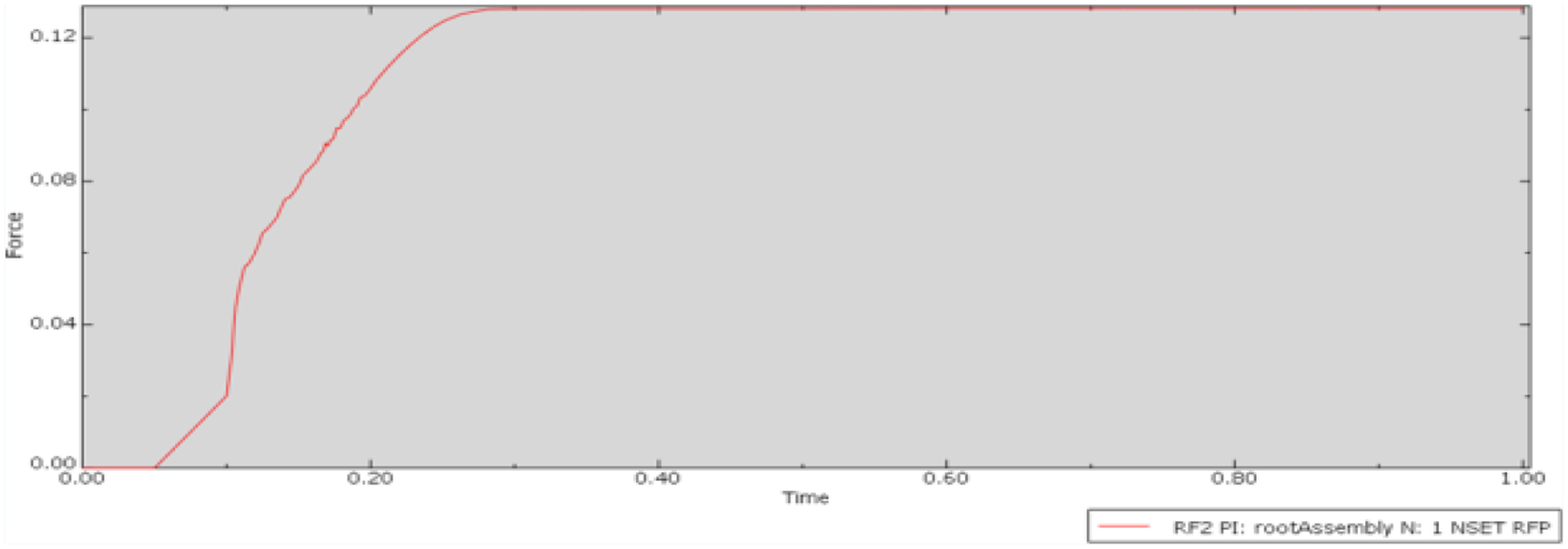
Plot of friction force versus time for the Parallel 3- point condition.

The deformation maps for the Parallel 3-point condition (Figure 9), revealed a slightly altered pattern of interaction between the two objects. The outer and inner surfaces near the contact areas exhibited deformation, while the core of the guide remained fairly unchanged. According to the plot of friction force over the duration of this condition (Figure 10), the peak friction force was approximately 0.13N. This represents an even greater reduction compared to the previous two experimental conditions, with the peak friction force decreased by nearly 98% from the Control condition. These results highlight the significant impact of ridge orientation on friction force, suggesting that the parallel orientation is superior in reducing friction force between the two parts.

## DISCUSSION

This study was conducted to investigate the interaction between the biopsy template and biopsy needle with the aim of optimizing core biopsy procedures. The primary objective was to introduce the concept of modifying the needle guide configuration to reduce the friction force associated with the biopsy system, and quantitatively evaluate this reduction across different geometrical configurations.

The purpose of the Null Control condition was to ascertain whether an overlap between the needle and guide should be induced in the simulation. A physical needle guide would possess minor surface imperfections, resulting in several small contact points between the guide and needle. The outcome of the Null Control condition, with a friction force order of magnitude of 10^−9N, underscores that these subtle interactions cannot be accurately simulated in the software. For this reason, a consistent induced overlap between the needle and guide was necessary for each condition. To achieve this, a slight increase in the diameter of the needle was required to ensure uniformity across experimental conditions and mimic real-world scenarios as best as possible.

The Control condition served as a representation of the conventional needle guide currently utilized in practice. While the software couldn’t replicate the micro-texture, a slight overlap was implemented to acknowledge the resistance expected between the two surfaces, reflecting real-world conditions. Utilizing the Control condition as a baseline facilitates the comparison of experimental conditions, determining whether the anticipated reduction in friction force occurred. With a peak friction force of approximately 6.0N, the Control condition aligns with the margin specified by the derived equation for control parameters (i.e., 8.43 ± 2.53). This suggests that the simulation methodology accurately mirrored a genuine physical scenario. Given that the Control condition exhibited the greatest area of contact between the needle and guide, approximately 3.99mm^2 as calculated by the equation for the area of an open cylinder, it serves as a reference point against which to evaluate the impact of reduced contact area in experimental conditions. The expectation was that experimental conditions would yield lower friction forces due to their decreased contact area compared to the Control condition.

The Perpendicular 5-point condition produced a peak friction force of approximately 4.0N and the Perpendicular 3- point condition produced a peak friction force of approximately 1.2N. The data obtained from the Perpendicular 5- point and Perpendicular 3-point conditions aligns with the trend predicted by the derived equation, indicating that a lower number of contact points and consequently a smaller total contact area between the two surfaces leads to a decreased magnitude of associated friction force. Therefore, the subsequent experimental condition, Parallel 3-point, was conducted to determine the impact of a change in the orientation of the contact points on friction force. The Parallel 3-point condition yielded the smallest friction force of around 0.13N. The plots depicting friction force over time for all conditions exhibited a consistent trend, with friction force increasing as the needle was inserted into the guide until stabilization at a maximum value once the needle was fully in contact with the guide. The low value for the friction force of the Parallel 3-point condition was likely due to a decreased area of contact between the two part surfaces when compared to the other 3-point condition. The average friction force across all experimental conditions was approximately 1.71N, falling within the margin estimated by the derived equation (i.e., 3.09 ± 1.55). This close alignment between simulated and predicted values suggests that the simulation faithfully represents the physical scenario it aimed to simulate, reinforcing the credibility of the findings.

In the experimental conditions, the contact between each ridge transitions from line contact to area contact as the needle interacts with it, as a result of the deformation of the semicircular ridges. In the perpendicular conditions, the overlap between the two surfaces induces deformation in the ridges, causing the contact to assume the shape of a rectangle spanning the circumference of the inner surface of the guide rather than a line as if no overlap or deformation took place. The area of this contact (as described by Equation 2) can be computed by multiplying the circumference of the inner surface of the needle guide (representing the length of the rectangle) by the overlap between the two surfaces (representing the height of the rectangle)

(2) $$A_{⊥}=\pi \cdot d_{i}\cdot \Delta \mu$$

This conversion transforms the contact area from a one-dimensional line to a two-dimensional area. The area of contact for the Parallel condition is determined using the same principle, but the length of the needle guide (1mm) represents the height of the rectangular contact area, while the overlap between the two surfaces denotes the width (Equation 3).


(3) $$A_{∥}=l\cdot \Delta \mu$$

Based on the calculations, the area of contact for each perpendicular ridge is about 0.018mm^2, whereas the contact area for each parallel ridge is approximately 0.0045mm^2. These values can be used to calculate

**Figure 12:**
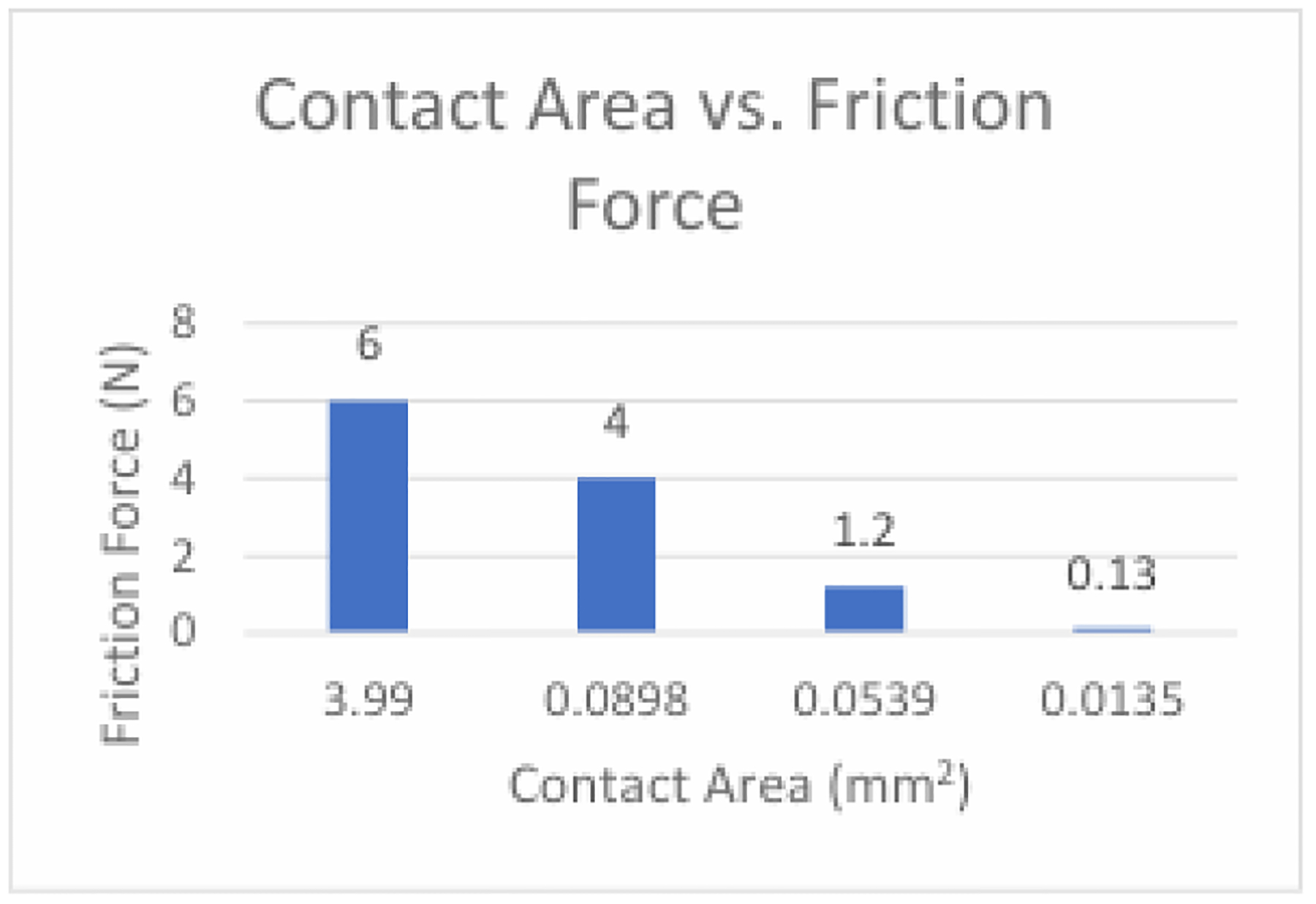
The value of the friction force compared to the total contact area of a condition. The lower the area of contact between two surfaces, the lower the friction force at their interface was found to be.

A plot can be constructed comparing the contact area versus the resulting friction force for the system (Figure 12).

The Parallel 3-point condition achieved a lower friction force due to the orientation of the ridges, which resulted in a reduced total contact area compared to the Perpendicular 3-point condition. Conversely, the Perpendicular 5-point condition exhibited the highest friction force, attributable to the increased number of contact points and subsequently greater contact area between the two surfaces compared to either of the two 3-point conditions. These findings highlight that the efficacy of the Parallel 3-point configuration in minimizing friction force between the needle and the guide was the greatest among all conditions. By considering the core biopsy template as an additional component of the biopsy procedure that may contribute friction to the system, friction reduction strategies focused on this aspect of the equipment can be explored. By mitigating friction across the biopsy procedure, the quality of biopsy cores can be substantially improved through reduced fragmentation, minimized needle deflection facilitated by higher insertion velocities, and decreased soft tissue deformation, enhancing the precision of targeting prostate lesions during biopsy procedures Achieving superior quality core biopsy specimens in fewer insertions minimizes the number of passes required, thereby reducing patient trauma and mitigating the risk of infection Additionally, the reduced friction force optimizes the histopathological quality of biopsy samples for accurate diagnosis and treatment planning. Ultimately, these advancements contribute to enhancing patient outcomes and overall procedural efficiency within the clinical setting.

## Figures and Tables

**Figure 11:**
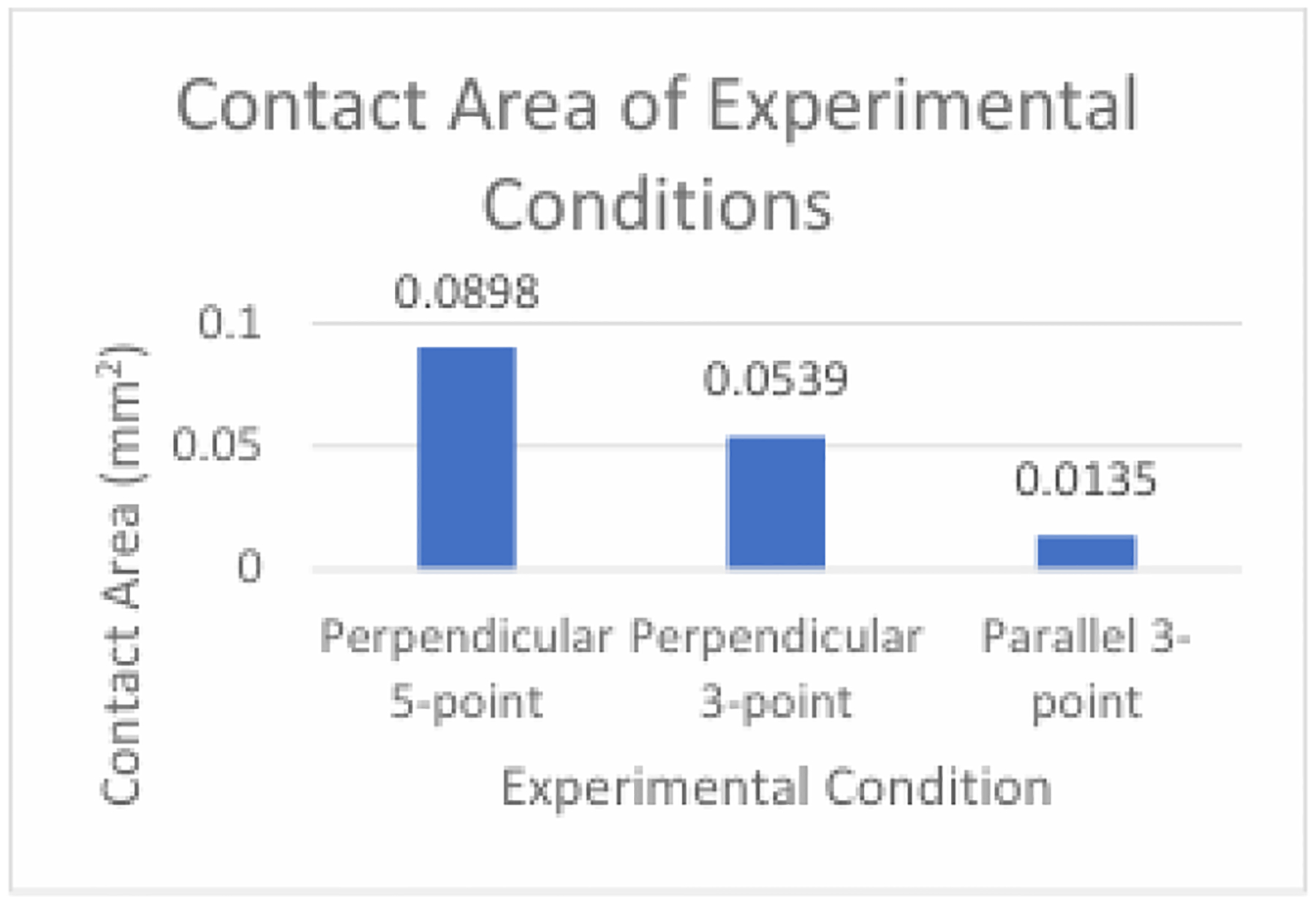
The total contact area of each condition. The 5-point condition had a greater contact area than both 3-point condition, and the parallel condition had a lower contact area than the perpendicular condition for the same number of contact points.

**Table 1:** Derived equation components. The fundamental equations (left) and variables (right) that were used for the derivation are shown

Fundamental Equations	Symbol	Variable
σ_rr_ ≈ 0	*P*_*i*_	Internal Pressure
$\sigma_{\theta \theta }=\frac{P_{i}(d_{i}+t)}{2t}$	σ_θθ_	Tangential stress
	σ_rr_	Radial stress
*σ* = *E*·*ε*	*d*_*i*_	Inner diameter
$\varepsilon =\frac{\Delta \mu }{r_{i}}$	*r*_*i*_	Inner radius
	*t*	Thickness
$r_{i}=\frac{1}{2}d_{i}$	***E***	Young’s Modulus
	μ	Coefficient of friction
*F* = μ·N(1+*f*_*A*_)	N	Normal force
$P=\frac{F}{A}$	*f*_*A*_	Area correction factor
*A* = *πd*_*i*_·*l*	Δμ	Overlap between contact surfaces

## References

[1] Siegel RL, Giaquinto AN, Jemal A. Cancer statistics, 2024. CA Cancer J Clin. 2024; 74: 12–49. 38230766 10.3322/caac.21820

[2] Grossman DC, Curry SJ, Owens DK, Bibbins-Domingo K, Caughey AB, Davidson KW, Screening for Prostate Cancer: US Preventive Services Task Force Recommendation Statement. JAMA. 2018; 319: 1901–1913. 29801017 10.1001/jama.2018.3710

[3] Schässburger KU, Paepke S, Saracco A, Azavedo E, Ekström C, Wiksell H. High velocity pulse biopsy device enables controllable and precise needle insertion and high yield tissue acquisition. Physica Medica. 2018; 46: 25–31. 29519406 10.1016/j.ejmp.2017.12.014

[4] Li W, Wang Y, Nteziyaremye V, Yamaguchi H, Shih AJ. Measurement of the Friction Force inside the Needle in Biopsy. J Manufact Sci Eng, Transactions of the ASME. 2016; 138.

[5] Delahunt B, Egevad L, Grignon DJ, Srigley JR, Samaratunga H. Prostate cancer grading: Recent developments and future directions. BJU Int. 2016; 117: 7–8. 10.1111/bju.13467 27094970

[6] Zhou SR, Chang E, Patankar A, Huang J, Marks LS, Natarajan S. Prostate Cancer Detection Rate of Freehand versus 3- Dimensional Template Mapping Biopsy Using a Magnetic Resonance Imaging-Ultrasound Fusion Device in Biopsy Naïve Men. J Urol. 2020; 203: 699–705. 31596671 10.1097/JU.0000000000000587 PMC7384745

[7] McGill CS, Schwartz JA, Moore JZ, McLaughlin PW, Shih AJ. Precision grid and hand motion for accurate needle insertion in brachytherapy. Med Phys. 2011; 38: 4749–4759. 21928648 10.1118/1.3611040

[8] Brede CM, Douville NJ, Jones S. Variable correlation of grid coordinates to core location in template prostate biopsy. Curr Urol. 2012; 6: 194–198. 10.1159/000343538 PMC3783329 24917742

[9] Gao D, Lei Y, Yao B. Analysis of Dynamic Tissue Deformation during Needle Insertion into Soft Tissue. IFAC Proceedings Volumes. 2013; 46: 684–691.

[10] Casanova F, Carney PR, Sarntinoranont M. In vivo evaluation of needle force and friction stress during insertion at varying insertion speed into the brain. J Neurosci Meth. 2014; 237: 79–89. 10.1016/j.jneumeth.2014.08.012 PMC8011429 25151066

[11] Schässburger KU, Paepke S, Saracco A, Azavedo E, Ekström C, Wiksell H. High velocity pulse biopsy device enables controllable and precise needle insertion and high yield tissue acquisition. Physica Medica. 2018; 46: 25–31. 29519406 10.1016/j.ejmp.2017.12.014

[12] Patra A, Keshava SN. Biopsy with Side-Cutting Coaxial Needle-Knowing the Cutting Length and Throw Length. Ind J Radiol Imaging. 2021; 31: 933–938. 10.1055/s-0041-1741050 PMC8817798 35136506

[13] Franiel T, Fritzsche F, Staack A, Rost J, Hamm B, Beyersdorff D. Histopathologische qualität von prostatastanzzylindern: Vergleich einer MR-kompatiblen biopsienadel mit einer im ultraschall eingesetzten ferromagnetischen biopsienadel. RoFoFortschritte auf dem Gebiet der Rontgenstrahlen und der Bildgebenden Verfahren. 2006; 178: 1212–1218. 10.1055/s-2006-926936 16933199

[14] Retlaw Industries. Types of Plastic Overview: What are the Materials Used in Plastic Injection Molding? 2024.

[15] SpecialChem SA. Modulus of Elasticity. 2024.

[16] KYOCERA SGS Precision Tools Europe Ltd. Titanium Properties. 2024.

